# Evaluation of the childhood obesity prevention program Kids - 'Go for your life'

**DOI:** 10.1186/1471-2458-10-288

**Published:** 2010-05-28

**Authors:** Andrea de Silva-Sanigorski, Lauren Prosser, Lauren Carpenter, Suzy Honisett, Lisa Gibbs, Marj Moodie, Lauren Sheppard, Boyd Swinburn, Elizabeth Waters

**Affiliations:** 1 The McCaughey Centre, Melbourne School of Population Health, The University of Melbourne, Victoria, Australia; 2 The Cancer Council Victoria and Diabetes Australia, Victoria, Australia; 3 The WHO Collaborating Centre for Obesity Prevention, Faculty of Health, Medicine, Nursing and Behavioural Sciences, Deakin University, Victoria, Australia; 4 Deakin Health Economics, Deakin University, Victoria, Australia

## Abstract

**Background:**

Kids - 'Go for your life' (K-GFYL) is an award-based health promotion program being implemented across Victoria, Australia. The program aims to reduce the risk of childhood obesity by improving the socio-cultural, policy and physical environments in children's care and educational settings. Membership of the K-GFYL program is open to all primary and pre-schools and early childhood services across the State. Once in the program, member schools and services are centrally supported to undertake the health promotion (intervention) activities. Once the K-GFYL program 'criteria' are reached the school/service is assessed and 'awarded'. This paper describes the design of the evaluation of the statewide K-GFYL intervention program.

**Methods/Design:**

The evaluation is mixed method and cross sectional and aims to:

1) Determine if K-GFYL award status is associated with more health promoting environments in schools/services compared to those who are members only;

2) Determine if children attending K-GFYL award schools/services have higher levels of healthy eating and physical activity-related behaviors compared to those who are members only;

3) Examine the barriers to implementing and achieving the K-GFYL award; and

4) Determine the economic cost of implementing K-GFYL in primary schools

Parent surveys will capture information about the home environment and child dietary and physical activity-related behaviors. Environmental questionnaires in early childhood settings and schools will capture information on the physical activity and nutrition environment and current health promotion activities. Lunchbox surveys and a set of open-ended questions for kindergarten parents will provide additional data. Resource use associated with the intervention activities will be collected from primary schools for cost analysis.

**Discussion:**

The K-GFYL award program is a community-wide intervention that requires a comprehensive, multi-level evaluation. The evaluation design is constrained by the lack of a non-K-GFYL control group, short time frames and delayed funding of this large scale evaluation across all intervention settings. However, despite this, the evaluation will generate valuable evidence about the utility of a community-wide environmental approach to preventing childhood obesity which will inform future public health policies and health promotion programs internationally.

**Trial Registration:**

ACTRN12609001075279

## Background

Currently, there is only limited evidence available about effective strategies to prevent childhood obesity. However community-based approaches to prevent childhood obesity that are multi-sector and multi-strategy show promise as being effective and also have the potential to be equitable, sustainable and cost-effective [1-8]. Schools, preschools and child care settings have all been identified as important settings for population-based obesity prevention efforts given their important role in promoting healthy eating and physical activity for children [9-11].

To date, settings-based child obesity prevention interventions have primarily been conducted in schools [1]. However, the socio-ecological framework identifies the multi-level influences on individual behaviors and recognizes that culture and ethos of the settings, organizational policies, practices and regulations, and engagement with the wider community are all important factors [12]. Kids - 'Go for your life' (K-GFYL) is a settings-based health promotion intervention that aims to reduce the risk of childhood obesity by using an award-based program to improve the socio-cultural, policy, and physical environments related to healthy eating and physical activity across the community [13].

### Kids 'Go for your life' Award Program

**Figure 1:**
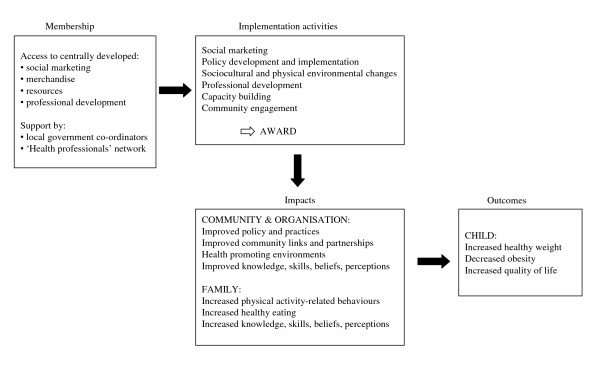
**Kids-Go for your life program logic model**.

The K-GFYL intervention is being implemented in a range of children's settings, including primary (elementary) schools (children aged five to twelve years), pre-school (also known as kindergarten; for children aged three to five years), and the early childhood services family day care (FDC, a home-based child care service for children from birth to twelve years) and long day care (LDC, centre-based care for children aged from birth to five years). The K-GFYL program includes support and professional development for staff within those settings to make policy and practice changes that promote healthy eating and physical activity for children and their families [13]. The key obesity-related behaviors targeted are: increasing fruit, vegetable and water consumption; reducing consumption of foods high in fat, salt and sugar and sweet drinks; increasing participation in physical activity; reducing sedentary behavior (such as screen time); and increasing active transport [13]. The intervention was designed within a Health Promoting Schools (HPS) framework [14]; which requires a whole of school/service approach to health promotion. K-GFYL is an award-based program and membership is open to all primary and pre-schools, and early childhood services across the state of Victoria, Australia. Once members, the schools and services are supported to implement the program by a state-wide coordination team, a local government coordinator (available in 10 of the 79 local government areas in Victoria) and local community members of the Kids - 'Go for your life' Health Professionals' network. Once the school or service deems it has fully implemented the program and achieved all of the policy and practice requirements, they apply to become 'awarded' as a Kids - 'Go for your life' school or service. The application is reviewed and assessed by the state-wide coordination team and the award conferred if all of the appropriate criteria are met. From that time, they are referred to as a K-GFYL 'awarded' school or service. It is hypothesized that reaching award status increases children's physical activity and healthy eating behaviors through the creation of health promoting environments, capacity-building and community engagement (see Figure 1).

This paper describes the design of the evaluation of the K-GFYL intervention. Specifically the evaluation aims to:

1) Determine if K-GFYL award status is associated with more health promoting environments in schools/services compared to those who are members only;

2) Determine if children attending K-GFYL awarded schools/services have higher levels of healthy eating and physical activity-related behaviors compared to those who are members only;

3) Examine the barriers to implementing and achieving the K-GFYL award; and

4) Determine the economic cost of implementing K-GFYL in primary schools

## Methods/Design

### Evaluation design

When determining the appropriate evaluation design, the research team considered a number of contextual and limiting factors. These included 1) the implementation of the intervention program across the entire State and current high-level recruitment drive; 2) the funding of this evaluation more than 12 months after state-wide implementation began; 3) the requirement for the evaluation data to be available in a short time frame (all data was to be collected within one school term of 12 weeks); and 4) the limited evaluation funding for this large-scale impact and outcome evaluation in all of the settings targeted by the intervention. Taking these factors and the study aims into consideration, a mixed method, cross sectional evaluation design was deemed the most appropriate to determine differences in impact and outcome measures between those settings/services that were K-GFYL members only and those that were K-GFYL awarded. In the school setting, there will be two groups of awarded schools: newly awarded (<12 months) and longer term awarded (≥12 months), to enable an examination of the sustainability of program impacts after the award is conferred.

The K-GFYL intervention design, implementation strategies and materials were also carefully reviewed to determine the best evaluation methods given the constraints already mentioned. A summary of the main intervention activities implemented is provided in Table 1. Consistent with the intervention design, the evaluation design is guided by the socio-ecological framework [15] and accepted best practice for the evaluation of health promotion projects of this kind [16] (see Table 2). The evaluation was therefore also multi-level and the instruments are outlined in Table 2. The evaluation approach varies across settings due to the nature of the setting, data collection costs and accessibility to the setting/service and participants.

**Table 1:** K-GFYL health promotion activities implemented in settings

K-GFYL Program area	Health promotion activity
Water and sweet drinks	Children have access to water throughout the day (water bottles, jugs and fountains)
	Sweet drinks are restricted or not allowed
	Having access to drinking water is reflected in school/service policy
Fruit and Vegetables	Defined periods of time when students/children are encouraged to eat fruit and vegetables only (e.g. Fruit & Veg play lunch, fruit break)
	School/service policy that includes a fruit break
	Information is provided to parents about healthy lunches and snacks
	Special days to promote fruit and vegetables such as: Free Fruit Fridays, nude food days (e.g. no packaged snacks), apple slinky days
	Students/children are involved in activities to grow and cook food
	Staff role modeling healthy eating and drinking practices
	Establishing links with local fruit and vegetable retailers
	Food is not used as a reward, incentive or for comfort
Unhealthy food and drinks	The school/service policy restricts unhealthy and promotes healthy foods and drinks
	The food service provides foods consistent with healthy eating guidelines and government policy
	Fundraising is consistent with healthy eating guidelines and policies
Physical Activity	Professional development opportunities for staff
	Established partnerships with local community and physical activity organizations^1^
	Structured and free active play sessions are planned on a daily basis
	Children have at least 30 minutes of structured active play and at least 60 minutes (and up to several hours) of unstructured play during their care session^2^
	Structural equipment (e.g. sandpit, fixed play equipment) is available for all students
	Classroom programs encourage physical activity during break time for all students^1^
	Staff role model being physically active
	Physical activity/active play policies are implemented
	Restrictions on screen-based activities (e.g. TV, DVDs, computers)
	Parents are provided with information about screen time recommendations for children and children's physical activity
Safe and Active	Promotion of walking/riding to school or other places ≥once/term^1^
Transport	Available bike storage for student and staff
	Child cyclist and pedestrian safety program
	Traffic calming measures outside the school/service
Curriculum and Policy	Teaching focused on healthy eating and physical activity, is incorporated in to the school curriculum plan^1^
	Whole school/service approach to healthy eating and physical activity
Families and School Community	Parents/carers are provided with information about the healthy eating and physical activity policy requirements and/or copies of the policies
	Information sessions/workshops for parents on healthy eating and physical activity

^1^Activities related to primary school settings only; ^2^Activities related to care settings only

**Table 2:** Overview of the evaluation design and instruments incorporating the socio-ecological framework [13]

Level of change	Description	Instrument	Participants	Setting
Environment/Policy	Policies, advocacy, environments, structures	Environment assessment Policy assessment	Settings staff	All
Intrapersonal	Individual characteristics/behaviours	Lunch Box Survey	Staff report of children's lunchbox content	PreS, FDC
		Child Health Questionnaire	Parents/guardians	PS
Community	Shared identities, experiences and resources for health	Qualitative data	Parents/guardians	PreS, PS

PreS: Preschools; FDC: Family Day Care; PS: Primary school;

### Sampling

#### Primary Schools

Given the constraints discussed above, a logistically feasible sample size is estimated to be 80 schools. This will be stratified as 30 member, 30 'newly-awarded' (<12 months) and 20 'long term awarded' (≥12 months) schools. A random sample will be drawn from within each strata, however to ensure consistency across the sample and simplify the study design, sampling will be restricted to Government schools (80% of the K-GFYL schools). In addition, for the 'member' group only schools that have recently (< 3 months) joined K-GFYL will be sampled, in an attempt to recruit schools that are not well advanced with the implementation and therefore maximize differences between the groups. With a conservative estimate of intraclass correlation (ICC) of 0.02, and sampling 20-30 students per school (design effect of at most 1.98), for interval behavioral data this sample size will detect a difference of 0.26 based on the example of fruit intake with a mean of 1.99 serves and a standard deviation of 1.14. For data that are categorical, this sample size allows us to detect medium effect sizes, specifically a difference of 10 percentage points between member and awarded services, with 80% power at a significance level of 0.05. Given reasons of cost and concern regarding respondent burden on schools, the sample for the resource use questionnaire will be restricted to 10 schools in each of the three groups.

#### Preschools and early childhood settings

Given the time and funding constraints a logistically feasible sample size is deemed to be 50 preschools and services; stratified as 20 member and 30 awarded for preschools and the LDC services. For continuous data, this sample size will detect a difference of 1.0 unit for a variable such as staff ratings of support for health promotion activities, with a mean of 8.4 and a standard deviation of 1.3, with 80% power and significance level of 0.05. For data that are categorical, this sample size provides the ability to detect large effect sizes, specifically a difference of 40 percentage points between member and awarded services, with 80% power at a significance level of 0.05. At the time of planning, thirty FDC services are members and awarded in the K-GFYL program and each of these services will be invited to participate in the evaluation.

A set of open-ended questions will be given to preschool parents (from 5-7 participating kindergartens) to examine their experiences with the K-GFYL intervention. This sample will be purposively drawn from the participating kindergartens to include member and awarded kindergartens from socio-economically disadvantaged areas and where possible, from at least one area with a high culturally and linguistically diverse population.

### Data Collection Instruments

The *School Environment Questionnaire (SEQ) *is a 129 question instrument designed to examine key elements of the school environment. The questionnaire contains items that have been used previously in similar studies we have conducted [17-19] and forms the structure for key informant interviews to capture physical (eg. adequacy of sporting and active play equipment), policy (eg. adoption of nutrition and physical activity policies) and socio-cultural (eg. teachers' role modeling physically active behaviors) elements of the school environment. This structured interview is conducted with 2-3 members of the school staff and also captures information related to the following areas: general demographic information; the food service; school food/nutrition policy(ies); the nutrition environment; school physical activity policy(ies); the physical activity environment; staff knowledge, skills and attitudes; and program implementation activities. Interviews will be completed in a single session approximately 45 minutes in duration and will be conducted by trained research staff. For each question one response will be recorded and where there is disagreement, staff will be asked to discuss the issue until consensus is reached.

The *Child Health Questionnaire *contains 51 items and is designed to capture information from parents/carers of primary school children regarding their child's physical activity, sedentary and nutrition related behaviors. The majority of survey items have previously been used by our research group in similar studies [3,8,17,19,20]

The *Economic Resource Questionnaire *is designed to determine what resources the school has used to implement activities for the K-GFYL intervention. It is to be completed by the staff member who has the most knowledge about these activities and in most cases, this will be the same staff member(s) who complete the SEQ (above). The specific activities reported on will be extracted from the SEQ and will reflect school activities undertaken in the previous three school months. For each activity, the following details are required: a brief description of the activity, frequency of activity, target group (eg. whole of school or a particular class), the category of costs (personnel time, equipment, food and drink, other resources), quantity, provider of the resource (school, parents, external sponsor, grant etc), and the actual cost (if known). This questionnaire is for use in primary schools only and the data will be collected either through a face-to-face or telephone interview.

The *environmental questionnaires *used in the preschool and early childhood settings are similar to the SEQ and have been used previously by our research group for similar studies [17]. Whilst the questionnaire is consistent across settings, it is adapted to the nature of each of the services (FDC, LDC and preschool) to be examined. The questionnaire comprises 12 sections, containing approximately 244 items in total and captures the following: general demographics; food service; children's daily activities; staff knowledge; skills and attitudes; communication strategies; physical environment; policy environment; and intervention activities.

A child *lunch box survey *will also be conducted in preschools and the FDC services. These settings were chosen as children bring lunch and snacks with them each day in both settings and because of previous use of the survey in these settings. The survey is observational and designed for use by the teacher/carer who records the number of children bringing items from a range of food/drink groups for lunch, snack or both (incidence reporting). The food groupings are: fresh fruit and vegetables; packaged snack foods; high fat/high sugar snack foods; healthy snacks; sandwiches/rolls with high sugar filling; sandwiches/rolls with healthy fillings; and chocolates/sweets/lollies. The drink categories are: water, sweet drinks, plain milk, other.

#### Policy analysis

Two policy checklists were developed for this evaluation based on our existing knowledge of school and service nutrition and physical activity policies and current evidence about important policy elements to promote nutrition and physical activity in children's settings [21-23]. The checklists have been piloted with a random sample of schools and services to ensure they extract the information required consistently across policies. After some refinement of wording, these checklists have been finalized (below) and will be used by trained research staff to determine the elements contained in the nutrition and physical activity policies. Research staff will also provide a rating (very poor, poor, good, very good) of 1) the structure and layout; and 2) readability and ease of understanding (simple and clear) of each policy.

Policy elements for nutrition/food-related policies:

1. Limit or restrict foods and drinks available through the food service (internal/external)

2. Ensure the food service menu meets government guidelines for healthy eating or nutritional quality

3. Ensure the availability of water for students/children

4. Restricting students' access to stores and food outlets, schools only

5. Restricting vending machines on the premises, schools only

6. Restricting foods associated with fundraising

7. Restricting foods associated with special events (e.g. birthdays, functions)

8. Setting aside adequate time for children to eat lunch/snacks

9. Promoting or restricting the types of foods that may be brought from home

10. Teaching that is focused on food and nutrition in the curriculum

11. Distribution of information to parents about healthy food and eating

12. Staff acting as role models in the area of healthy eating

13. Encouraging children to adopt healthy eating behaviors

14. Operating the food service on a not-for-profit basis

15. Foods are not used as rewards or punishment

16. Designated fruit/vegetable breaks

17. Promoting children's participation in growing, preparing and/or cooking food

18. Importance of healthy eating on learning outcomes (eg reduced absences, better behavior), schools only

19. Engaging with allied health professionals to implement health promotion activities

20. Providing professional development for staff regarding healthy eating

Policy elements for physical activity-related policies:

1. Providing access to play equipment out of class/session time

2. Promoting active transport to and from school/service

3. Planned teaching focused on active play

4. Providing information to parents about children's physical activity/active play

5. Promote active play/physical activity outside of service/school

6. Planned activities during school/session times

7. Promote a positive attitude toward active play and physical activity

8. Offer challenging and varied physical activities for children

9. Description of resources available for students to engage in physical activity (e.g. equipment, challenges, excursions)

10. Providing professional development for staff related to active play/physical activity

11. Ensuring that all children are active most of the time during physical activity sessions

12. Active play/physical activity is inclusive of all children, no matter ability or level

13. Ensuring children have access to water during active play and at all times

14. Encouraging participation in sport and physical education

15. Planned break time (e.g recess, lunchtime) physical activities

16. Ensuring the time allocated for formal physical education is consistent with recommended or mandated physical education times

*Open-ended question *data will be collected from kindergarten parents to explore their awareness of healthy eating and physical activity kindergarten activities; the challenges parents face around healthy eating and physical activity for their child and family; and additional comments on their child and family eating and physical activity habits.

### Data Analysis

Data collected will be entered into Stata 10.1 for cleaning and analysis. Preschool, primary school and LDC environmental data that is categorical in nature will be analyzed using Chi-squared or Fisher's exact tests depending on sample size. Data that is continuous or interval in nature (eg staff ratings, frequency of actions etc) will be analyzed using t-tests if normally distributed or by the Kruskal-Wallis equality-of-populations rank test if non-normally distributed. Analysis of the child behavioral data will be at the school level (school as the Primary Sampling Units, PSUs, using the *svy *commands in Stata) using logistic regression (binary and ordinal), and generalized linear models regression with a Poisson distribution for interval data. Child behavioral data will be adjusted for child age, gender and maternal education (as an indicator of socio-economic status). Analysis of the FDC data will be at the local government area level (as the PSUs). Continuous or interval impact and outcome data will be compared using the Wald test of difference in group means, and data that is categorical or ordinal will be analyzed using logistic regression and the design corrected chi squared statistic. An inductive thematic analysis will be conducted for qualitative data.

### Consenting and Ethics

All data is to be collected from adult participants, who will be asked to provide written informed consent prior to data collection. School principals, preschool directors and service managers will also provide consent before staff and parents are invited to participate. Approval for this study has been obtained from the University of Melbourne Human Research Ethics Committee (HREC #0828812) and the Department of Education and Early Childhood Development.

## Discussion

As a result of government policy and funding, the K-GFYL intervention was developed and implemented in Victoria, Australia. The award-based program utilizes socio-ecological and health promoting schools approaches to prevent childhood obesity across the entire community by targeting a range of children's settings and services. Currently there is no evidence available about the efficacy or effectiveness of such an approach for childhood obesity prevention, although there is a growing body of evidence that community-based approaches can be effective. Evaluations of community-based health promotion programs are challenging [16], however added challenges for this evaluation are the delayed and limited funding of this phase of the evaluation (relative to the start of the intervention and the collection of process evaluation and pilot data), the large-scale, state-wide implementation of the program (and therefore lack of available control group), the limited time available for data collection, and the lack of any local level population monitoring data (which limits our ability to determine the impact of the intervention of child anthropometric and weight status outcomes).

Despite these constraints and limitations we have planned a comprehensive evaluation that will generate valuable evidence about the impacts of the K-GFYL intervention in schools and early childhood services. This new evidence can inform future public health policies and the development and implementation of health promotion programs to prevent childhood obesity at a population level.

## Competing interests

The authors declare that they have no competing interests.

## Authors' contributions

All authors contributed to the study design and AdS-S and LP prepared the initial draft of the manuscript. All authors critically reviewed the manuscript and approved the final draft.

## Pre-publication history

The pre-publication history for this paper can be accessed here:

http://www.biomedcentral.com/1471-2458/10/288/prepub
